# SFRP2 Improves Mitochondrial Dynamics and Mitochondrial Biogenesis, Oxidative Stress, and Apoptosis in Diabetic Cardiomyopathy

**DOI:** 10.1155/2021/9265016

**Published:** 2021-11-08

**Authors:** Tianyi Ma, Xiaohui Huang, Haoxiao Zheng, Guolin Huang, Weiwen Li, Xinyue Liu, Jingjing Liang, Yue Cao, Yunzhao Hu, Yuli Huang

**Affiliations:** ^1^Department of Cardiology, Shunde Hospital, Southern Medical University, Foshan, China; ^2^Department of Cardiology, Affiliated Haikou Hospital of Xiangya Medical College, Central South University, Haikou, China

## Abstract

**Background:**

The mitochondrial dynamics and mitochondrial biogenesis are essential for maintaining the bioenergy function of mitochondria in diabetic cardiomyopathy (DCM). Previous studies have revealed that secreted frizzled-related protein 2 (SFRP2) is beneficial against apoptosis and oxidative stress. However, no research has confirmed whether SFRP2 regulates oxidative stress and apoptosis through mitochondrial function in DCM.

**Methods:**

Exposure of H9C2 cardiomyocytes in high glucose (HG) 25 mM and palmitic acid (PAL) 0.2 mM was used to simulate DCM *in vitro*. H9C2 cells with SFRP2 overexpression or SFRP2 knockdown were constructed and cultured under glucolipotoxicity or normal glucose conditions. An SD rat model of type 2 diabetes mellitus (T2DM) was generated using a high-fat diet combined with a low-dose STZ injection. Overexpression of SFRP2 in the rat model was generated by using an adeno-associated virus approach. CCK-8, TUNEL assay, and DHE staining were used to detect cell viability, and MitoTracker Red CMXRos was used to detect changes in mitochondrial membrane potential. We used qRT-PCR and western blot to further explore the mechanisms of SFRP2 regulating mitochondrial dynamics through the AMPK/PGC1-*α* pathway to improve diabetic cardiomyocyte injury.

**Results:**

Our results indicated that SFRP2 was significantly downregulated in H9C2 cells and cardiac tissues in T2DM conditions, accompanied by decreased expression of mitochondrial dysfunction. The mitochondrial membrane potential was reduced, and the cells were led to oxidative stress injury and apoptosis. Furthermore, the overexpression of SFRP2 could reverse apoptosis and promote mitochondrial function in T2DM conditions *in vitro* and *in vivo*. We also found that silencing endogenous SFRP2 could further promote glucolipotoxicity-induced mitochondrial dysfunction and apoptosis in cardiomyocytes, accompanied by downregulation of p-AMPK.

**Conclusion:**

SFRP2 exerted cardioprotective effects by salvaging mitochondrial function in an AMPK-PGC1-*α-*dependent manner, which modulates mitochondrial dynamics and mitochondrial biogenesis, reducing oxidative stress and apoptosis. SFRP2 may be a promising therapeutic biomarker in DCM.

## 1. Introduction

Diabetes and its complications have become a public health issue of great concern. The leading cause of death in diabetic patients is cardiovascular disease [1]. Our previous studies also showed that even if the blood glucose level is in the prediabetic population, the risk of long-term cardiovascular events and all-cause death is significantly increased [2, 3]. Diabetic cardiomyopathy (DCM) is a common cardiovascular complication of diabetes. It is considered the main factor for the high incidence and mortality of heart failure in diabetic patients [4].

Glucolipotoxicity stimulates myocardial cells to increase the production of reactive oxygen species (ROS), which is an important cause for the development of DCM [5–8]. Mitochondria are dynamic organelles that undergo frequent morphological changes through fusion and fission [4, 9]. Some studies have found that hyperglycemia and hyperlipidemia can influence mitochondrial dynamics and mitochondrial biogenesis [10, 11]. Glucolipotoxicity can cause a decrease in mitochondrial fusion and an increase in mitochondrial fission [12, 13], while inhibiting mitochondrial fission can reduce ROS production caused by high glucose [14].

Secreted frizzled-related protein 2 (SFRP2) is a secreted protein, which exists in the cell cytoplasm and cell matrix. SFRP2 has been shown to play a protective role in various cardiovascular diseases [15–17]. We have found that SFRP2 could reduce myocardial damage and myocardial cell apoptosis in vivo and in MI rat model [18]. Furthermore, we found that the level of SFRP2 was positively associated with myocardial fibrosis, evaluated by cardiovascular magnetic resonance [19]. However, it remains unclear whether SFRP2 is a risk factor or a protective compensatory marker of myocardial fibrosis. At present, there are few studies that involve the role of SFRP2 in regulating mitochondrial function, and it is still controversial whether the role of SFRP2 in mitochondrial fusion is positive or negative [20, 21]. Given the important role of mitochondrial fusion in diabetic cardiomyopathy, we speculate that SFRP2 may regulate and improve mitochondrial fusion to reduce cardiomyocyte damage caused by high glucose.

Therefore, the aims of the present study were (1) to clarify the molecular signaling of glucolipotoxicity that leads to impaired mitochondrial dynamics and mitochondrial biogenesis of cardiomyocytes and (2) to verify whether SFRP2 can reduce the oxidation stress, apoptosis, and mitochondrial dynamics and mitochondrial biogenesis of cardiomyocytes caused by glucolipotoxicity and illustrate the underlying mechanisms.

## 2. Materials and Methods

### 2.1. Cell Culture

The rat cardiomyocyte cell line H9C2 was purchased from the Cell Bank of Chinese Scientific Academy (Shanghai, China). H9C2 cells were cultured in DMEM (Gibco, USA) media supplemented with 10% fetal bovine serum (Gibco, USA) and 50 U/mL penicillin and 50 *μ*g/mL streptomycin. Cells were maintained in a standard humidified incubator at 37°C with 5% CO_2_. The culture media were changed every second day. We added 25 mM glucose and 0.2 mM palmitic acid (PAL, Sigma, USA) to the medium in the high-glucose group. 5 mM glucose was added to the cells in the control group. The cells were cultured for 48 h and used for further experiments.

### 2.2. Expression Vector and Transfection

The cDNA of rat *Sfrp2* was PCR-amplified and cloned into LV-003 lentivirus vector (Forevergen Biosciences Center, Guangzhou, China). According to the manufacturer's recommendations, transfection was performed using Lipofectamine™ 3000 reagent (Thermo Fisher Scientific, Waltham, MA, USA). The lentiviral vector and packaging vector were cotransfected into 293T cells to produce recombinant lentivirus. H9C2 cells were exposed to recombinant lentivirus and cultured in a medium containing 2 *μ*g/mL puromycin to generate H9C2-SFRP2 and an empty vector expressing H9C2 (H9C2-EGFP). To confirm the efficiency of transfection of SFRP2 lentivirus, H9C2-EGFP and H9C2-SFRP2 were used to detect by qPCR and western blot, respectively.

### 2.3. Cell Viability

Cell viability was tested by Cell Counting Kit-8 (CCK-8). The H9C2 cells were seeded in 96-well plates at a concentration of 3000 cells/well. The cells received treatment with 5 mM glucose or 25 mM glucose and 0.2 mM PAL for 24, 48, and 72 h. Subsequently, 10 *μ*L was added to each well of CCK-8 immediately, and the cells were incubated for 2 h at 37°C. The absorbance was read at 450 nm on a microplate reader.

### 2.4. Animal Model

An SD rat model of T2DM was generated using a high-fat diet combined with low-dose STZ injection [22–25]. Forty adult male Sprague–Dawley (SD) rats (220–250 g) received the adaptive feeding by two weeks. There were ten rats in the WT group which continued to receive a normal diet. The remaining rats were fed with a high-fat diet. After 4 weeks of feeding, the rats were intraperitoneally injected with 30 mg/kg STZ to induce a model of T2DM. Animals with fasting blood glucose (FBG) levels higher than 16.7 mmol/L three days after the STZ injection were considered as successful T2DM models. Adeno-associated viruses expressing SFRP2 or EGFP were ordered from Hanbio Biotechnology Co. Ltd. (Shanghai, China). After the diabetes model was built, ten experimental rats in the DCM-EGFP group were injected with AAV-EGFP and ten experimental rats in the DCM-SFRP2 group were injected with AAV-SFRP2 via the tail vein, resulting in a model of control group or overexpression of SFRP2. The ten experimental rats left were the DCM group. The efficiency of virus infection and the expression of SFRP2 in rats were further confirmed by qPCR.

### 2.5. Hematoxylin-Eosin (HE) or Masson's Trichrome Staining

Cardiac tissue of each group was isolated from rats and fixed with 4% paraformaldehyde. The tissue was embedded in paraffin and cut into sections (4 *μ*m). Then, we observed the pathological and morphological changes of the myocardial tissue of each group by hematoxylin-eosin (HE) under an optical microscope (Olympus, Tokyo, Japan). Masson's trichrome staining of paraffin-embedded cardiac tissue from rats was performed to examine the myocardial fibrosis. The degree of the myocardial fibrosis in Masson's trichrome staining sections was determined using ImageJ software.

### 2.6. TUNEL Assay

H9C2 cells or cardiac ventricle tissues were analyzed for apoptosis with terminal transferase UTP nick end labeling (TUNEL) assay. The H9C2 cells in the 12-well plate or cardiac ventricle tissues were washed with PBS and fixed with 4% paraformaldehyde for 30 minutes. After being washed with PBS, the samples were added 0.3% Triton X-100 PBS mixture and incubated at room temperature for 5 min. Then, we added 50 *μ*L of TUNEL detection solution prepared in advance to the samples, and they were incubated for 60 min at 37°C in the dark. Further, we added DAPI to mount the slide and observe under a fluorescence microscope.

### 2.7. Measurement of ROS Level

To assess ROS of the H9C2 cells, a ROS Assay Kit (Beyotime Biotechnology, China) was used to detect intracellular reactive oxygen species. H9C2 cells were incubated with 5 *μ*M DHE at 37°C for 30 min in a dark incubator. Then, fluorescence microscopy was used to examine the fluorescence.

### 2.8. Western Blot

Western blot analysis was performed as described previously [18, 26]. Total proteins from the H9C2 cells and cardiac tissue of rats were lysed by the RIPA buffer (Beyotime, P0013B) according to the manufacturer's instructions. Protein concentration was determined using a BCA Protein Assay Kit (Beyotime, P0011). After being boiled, protein lysates (30 *μ*g protein per sample) were loaded. Proteins were separated by sodium dodecyl sulfate polyacrylamide gels (SDS-PAGE) and transferred onto the PVDF membrane. The membranes were blocked with 5% fat-free milk. After washing, the membranes were probed with primary antibodies overnight at 4°C. After incubation with their corresponding secondary antibody for 1 h at room temperature, protein bands were visualized with chemiluminescence (ECL; Forevergen Biosciences Center, Guangzhou, China). Blots were quantified by densitometric scanning. The level of protein expression was normalized against actin controls.

### 2.9. Quantitative Real-Time PCR

Myocardial gene expression of *Sfrp2* was performed by quantitative real-time PCR (qRT-PCR). Total RNA was extracted using the TRIzol reagent (Life Technologies). The RNA samples reverse-transcribed to cDNA using an RNA reverse were transcribed with Maxima First Strand cDNA Synthesis Kit (Takara, Dalian, China). The primers used were as follows: forward 5′-CATGGGACAGAAACAGGGTGGA-3′ and reverse 5′-GAGGTCGCAGAGTGGAAGTGGT-3′ for *Sfrp2*; forward 5′-GTATCGGACGCCTGGTTAC-3′ and reverse 5′-ACTGGAACATGTAGACCATGTAGTT-3′ for *GAPDH*.

### 2.10. Mitochondrial Membrane Potential

MitoTracker Red CMXRos (mitochondrial red fluorescent probe) can specifically label the biologically active mitochondria in the cell and detect the mitochondrial membrane potential (MMP). The cells were spread in a 12-well plate and treated with normal glucose or HG and PA for 48 h. After treatment, the plate was incubated in MitoTracker Red CMXRos staining solution (0.2 *μ*M) at 37°C for 30 min. Subsequently, the plates were replaced with the fresh cell culture medium. A fluorescence microscope was used to observe the cells. The MMP was calculated as the fluorescence intensity ratio.

### 2.11. Immunofluorescence Staining

For immunofluorescence staining, cells were placed on culture slides, fixed by 4% paraformaldehyde for 30 min, then permeated with 0.1% Triton X-100 for 30 min, and blocked with 2% BSA for 60 min at room temperature. Subsequently, the primary antibodies were incubated at 4°C overnight, followed by incubation with secondary antibodies for 60 min at room temperature. Nuclei were counterstained with DAPI. Finally, the images were examined with a fluorescence microscope.

### 2.12. Statistical Analysis

All experiments were performed at least three times. Data were analyzed using GraphPad Prism v.6 (GraphPad Software Inc., La Jolla, CA, USA). The data are presented as mean ± SD. We used Student's *t*-test to analyze the differences between the groups. A value of *P* < 0.05 was considered to indicate statistically significant differences between groups.

## 3. Results

### 3.1. Glucolipotoxicity Increased Oxidative Stress and Promoted Apoptosis in H9C2 Cardiomyocytes

**Figure 1:**
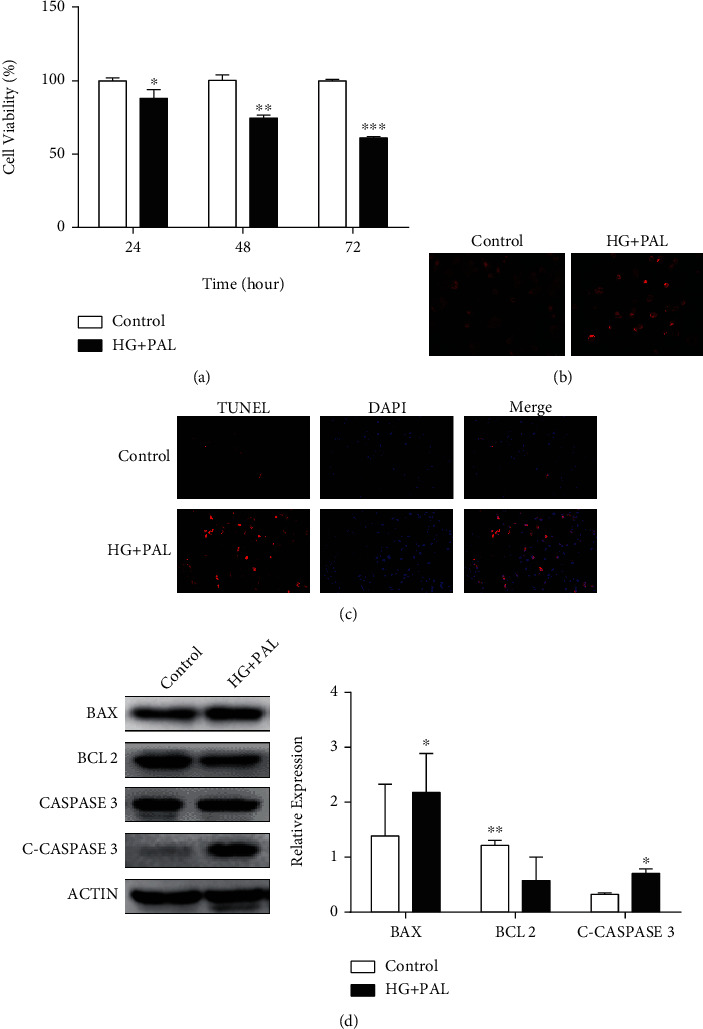
Glucolipotoxicity increased oxidative stress and promoted apoptosis in H9C2 cardiomyocytes. (a) Cell viability of H9C2 cardiomyocytes, treated with 25 mM glucose and 0.2 mM PAL for 24, 48, and 72 h, was measured by CCK-8. (b) The H9C2 cardiomyocytes were treated with indicated concentrations of HG and PAL for 48 h, and intracellular ROS was detected by DHE staining. (c) The apoptotic cells were detected by TUNEL assay. (d) Western blot analysis of BAX, BCL-2, CASPASE-3, and C-CASPASE-3 expressions in H9C2 cells treated with indicated concentrations of HG and PAL for 48 h. ^∗^*P* < 0.05; ^∗∗^*P* < 0.01; ^∗∗∗^*P* < 0.001.

To investigate the detrimental effect of glucolipotoxicity on H9C2 cells, a cohort of H9C2 cells was treated with 25 mM glucose and 0.2 mM PAL for 24, 48, and 72 h. As shown in Figure 1(a), H9C2 cells with HG and PAL exhibited decreased cell viability at every time point. Compared with normal conditions, an increase of ROS was observed in cells treated with glucolipotoxicity for 48 h (Figure 1(b)). Glucolipotoxicity for 48 h significantly increased cell death in cultured H9C2 cardiomyocytes (Figure 1(c)). BAX, BCL-2, CASPASE-3, and C-CASPASE-3 are apoptosis-related factors [27]. After glucolipotoxicity stimulation for 48 h, BAX and C-CASPASE-3 protein expressions were upregulated while BCL-2 was downregulated (Figure 1(d)). Taken together, these results demonstrated that glucolipotoxicity increased oxidative stress and promoted apoptosis in H9C2 cells.

### 3.2. Glucolipotoxicity Induced Mitochondrial Dysfunction in H9C2 Cardiomyocytes

**Figure 2:**
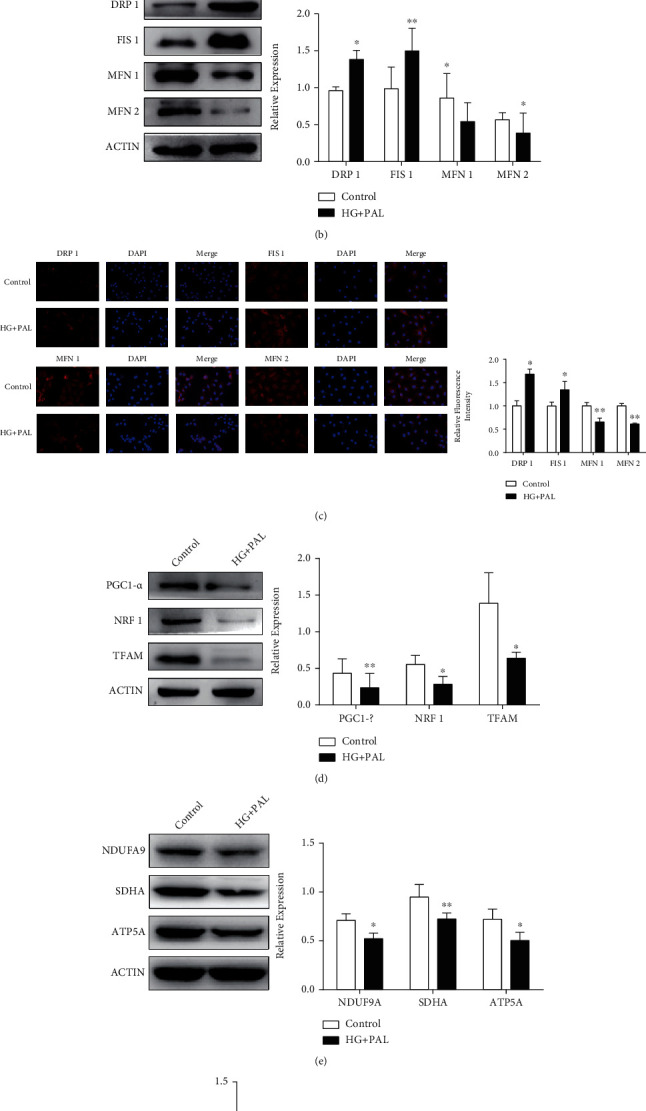
Glucolipotoxicity induced mitochondrial dysfunction in H9C2 cardiomyocytes. (a) The mitochondrial membrane potential levels of H9C2 cells treated with HG (25 mM) and PAL (0.2 mM) were evaluated by MitoTracker Red CMXRos. (b) The mitochondrial dynamics markers, DRP1, FIS1, MFN1, and MFN2, were detected by western blot analysis. (c) Immunofluorescence analysis was performed to detect the expression level of DRP1, FIS1, MFN1, and MFN2. (d) Western blot analysis of PGC1-*α*, NRF1, and TFAM expressions in H9C2 cells treated with HG and PAL for 48 h. (e) Mitochondrial respiratory chain proteins NDUFA9, SDHA, and ATP5A were detected by western blot. (f) The ATP level was analyzed. ^∗^*P* < 0.05; ^∗∗^*P* < 0.01.

Mitochondrial membrane potential levels of the H9C2 cells treated with glucolipotoxicity were evaluated by MitoTracker Red CMXRos [28]. In the glucolipotoxicity group, mitochondrial membrane potentials decreased, compared with the control (Figure 2(a)). As shown in Figures 2(b) and 2(c), the expression of the DRP1 and FIS1 protein was also increased progressively after treatment with glucolipotoxicity stimulation for 48 h, while the expression of MFN1 and MFN2 significantly decreased by western blot and immunofluorescence analysis. Furthermore, we found PGC1-*α* downregulated in a glucolipotoxic milieu, accompanied by a downregulation of NRF1 and TFAM (Figure 2(d)). We also detected mitochondrial respiratory chain protein and ATP. The level of NDUFA9, SDHA, and ATP5A and the level of ATP were decreased in the glucolipotoxic milieu. These findings confirmed that glucolipotoxicity stimulation could cause mitochondrial dysfunction in H9C2 cardiomyocytes.

### 3.3. Identification of SFRP2 as a Potential Regulator of DCM

**Figure 3:**
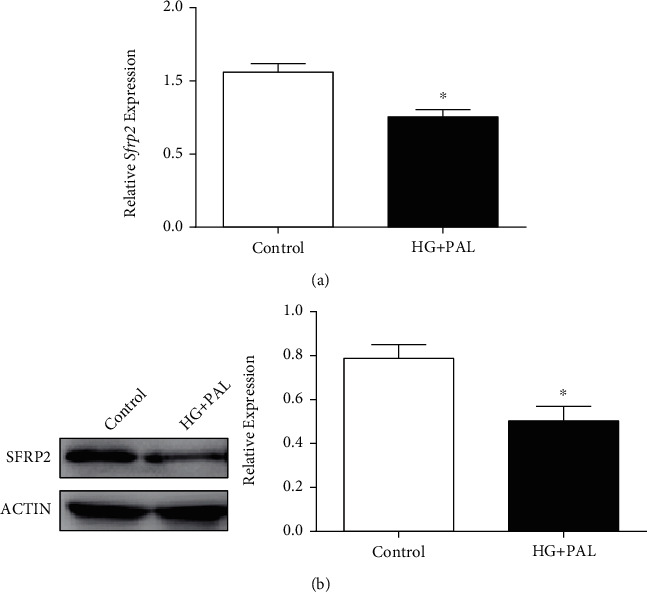
Identification of SFRP2 as a potential regulator of DCM. (a) qRT-PCR was used to detect *Sfrp2* in H9C2 cells treated with indicated concentrations of HG and PAL for 48 h. (b) Western blot was used to detect SFRP2 in H9C2 cells treated with indicated concentrations of HG and PAL for 48 h. ^∗^*P* < 0.05.

We measured the mRNA and protein levels of SFRP2, which might change in H9C2 cells exposed to glucolipotoxicity. The real-time PCR analysis revealed that the mRNA levels of *Sfrp2* significantly decreased in H9C2 cells with glucolipotoxicity (Figure 3(a)). Western blotting also showed a significant decrease in the level of the SFRP2 protein in the glucolipotoxic milieu. These results suggest that the level of SFRP2 protein was significantly reduced compared to that in the control group (Figure 3(b)).

### 3.4. Overexpression of SFRP2 Ameliorates Glucolipotoxicity-Induced Mitochondrial Dysfunction and Apoptosis in Cardiomyocytes

**Figure 4:**
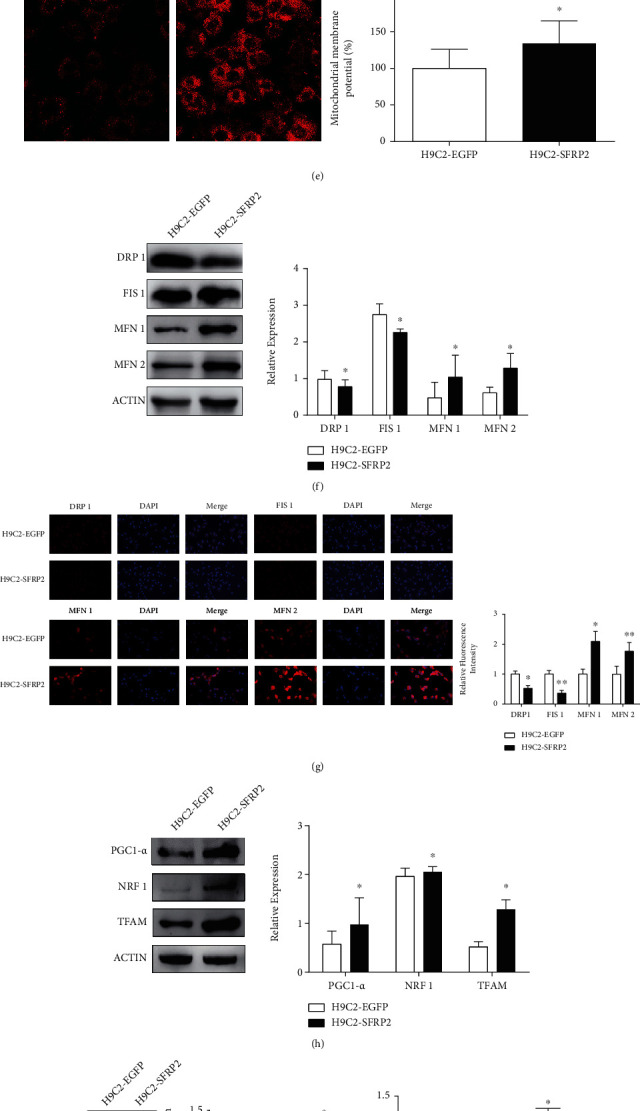
Overexpression of SFRP2 ameliorates glucolipotoxicity-induced mitochondrial dysfunction and apoptosis in cardiomyocytes. (a, b) qRT-PCR and western blot were used to detect the overexpression of SFRP2 in H9C2-EGFP and H9C2-SFRP2 cells. (c) Cell viability of H9C2-EGFP and H9C2-SFRP2, treated with 25 mM glucose and 0.2 mM PAL for 24, 48, and 72 h, was measured by CCK-8. (d) The intracellular ROS was detected by DHE staining. (e) The mitochondrial membrane potential levels of the H9C2-EGFP and H9C2-SFRP2 cells treated with the indicated concentrations of HG and PAL were evaluated by MitoTracker Red CMXRos. (f) Western blot analysis of the expression of DRP1, FIS1, MFN1, and MFN2 in H9C2-EGFP and H9C2-SFRP2 cells. (g) Immunofluorescence analysis was performed to detect the expression level of DRP1, FIS1, MFN1, and MFN2. (h) Expression levels of PGC1-*α*, NRF1, and TFAM were detected by western blot analysis. (i) The mitochondrial respiratory chain proteins NDUFA9, SDHA, and ATP5A were detected by western blot. (j) The ATP level was analyzed in H9C2-EGFP and H9C2-SFRP2 treated with the glucolipotoxic milieu. (k) The apoptotic cells were detected by TUNEL assay. (l) Western blot analysis of BAX, BCL-2, CASPASE-3, and C-CASPASE-3 expressions in H9C2-EGFP and H9C2-SFRP2 cells. (m) The expression of AMPK and p-AMPK was detected by western blot. ^∗^*P* < 0.05; ^∗∗∗^*P* < 0.001.

To clarify a causal relationship between SFRP2 and mitochondrial dysfunction and apoptosis in cardiomyocytes with glucolipotoxicity, we established an overexpression SFRP2 cell model and its control group and verified that the expression level of SFRP2 in H9C2-SFRP2 was higher than that in H9C2-EGFP cells (Figures 4(a) and 4(b)). Overexpression of SFRP2 can reverse the decrease in cell viability and reverse the oxidative stress damage caused by glucolipotoxicity (Figures 4(c) and 4(d)). To understand the function of mitochondria, we tested the changes in MMP and analyzed the expression of mitochondrial dynamics proteins after glucolipotoxicity treatment between H9C2-EGFP and H9C2-SFRP2 cells. The level of mitochondrial membrane potential in H9C2-EGFP was significantly lower than that in H9C2-SFRP2 (Figure 4(e)). H9C2-SFRP2 cells exhibited higher levels of MFN1 and MFN2 and lower levels of FIS1 and DRP1 compared to H9C2-EGFP (Figures 4(f) and 4(g)). The related indicators of mitochondrial biogenesis PGC1-*α* and TFAM in H9C2-SFRP2 with the glucolipotoxic milieu were significantly higher than those of the control group, accompanied by increased expression of NRF1 (Figure 4(h)). Meanwhile, the mitochondrial respiratory chain protein NDUFA9, SDHA, and ATP5A and the ATP level were increased in H9C2-SFRP2 than H9C2-EGFP (Figures 4(i) and 4(j)). As expected, SFRP2 overexpression improving apoptosis was observed by TUNEL assay and western blot analysis (Figures 4(k) and 4(l)). Furthermore, SFRP2 overexpression activated AMPK phosphorylation, which might be the mechanism by which SFRP2 improves mitochondrial function and apoptosis (Figure 4(m)).

### 3.5. Knockdown of SFRP2 Promoted Glucolipotoxicity-Induced Mitochondrial Dysfunction and Apoptosis in Cardiomyocytes

**Figure 5:**
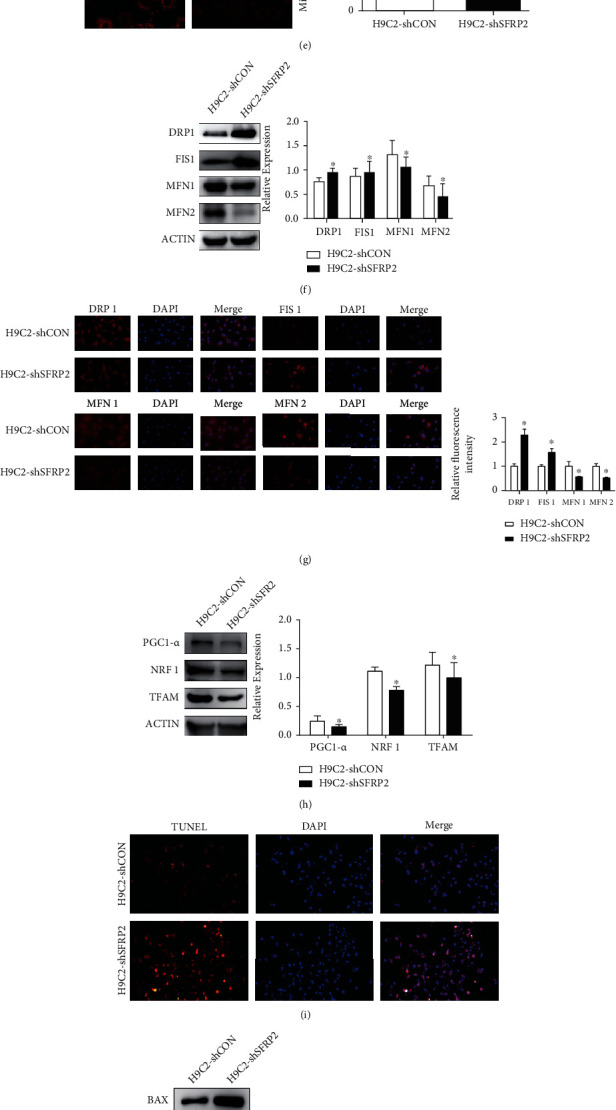
Knockdown of SFRP2 promoted glucolipotoxicity-induced mitochondrial dysfunction and apoptosis in cardiomyocytes. (a, b) qRT-PCR and western blot were used to detect the expression of Sfrp2 in H9C2-shCON and H9C2-shSFRP2 cells. (c) Cell viability of H9C2-shCON and H9C2-shSFRP2, treated with 25 mM glucose and 0.2 mM PAL for 24, 48, and 72 h, was measured by CCK-8. (d) The intracellular ROS was detected by DHE staining. (e) The mitochondrial membrane potential levels of the H9C2-shCON and H9C2-shSFRP2 cells treated with the indicated concentrations of HG and PAL were evaluated by MitoTracker Red CMXRos. (f) Western blot analysis of the expression of DRP1, FIS1, MFN1, and MFN2 in H9C2-shCON and H9C2-shSFRP2 cells. (g) Immunofluorescence analysis was performed to detect the expression level of DRP1, FIS1, MFN1, and MFN2. (h) Expression levels of PGC1-*α*, NRF1, and TFAM were detected by western blot analysis. (i) The apoptotic cells were detected by TUNEL assay. (j) Western blot analysis of BAX, BCL-2, CASPASE-3, and C-CASPASE-3 expressions in H9C2-shCON and H9C2-shSFRP2 cells. (k) The expression levels of AMPK and p-AMPK were detected by western blot. ^∗^*P* < 0.05; ^∗∗^*P* < 0.01.

To further verify the role of SFRP2 on promoting glucolipotoxicity-induced mitochondrial dysfunction and apoptosis in cardiomyocytes, we established H9C2-shCON and H9C2-shSFRP2 cells and confirmed the successful establishment of the cell model (Figures 5(a) and 5(b)). The results of CCK-8 showed that H9C2-shSFRP2 exhibited lower cell viability and more severe oxidative stress than H9C2-shCON after exposure to the glucolipotoxic milieu (Figures 5(c) and 5(d)). To investigate whether SFRP2 stimulates mitochondrial dynamics and mitochondrial biogenesis in cultured cardiomyocytes in response to glucolipotoxicity treatment, we also tested the MMP and the expression levels of mitochondrial fusion and fission proteins under the condition of knocking down SFRP2. Experimental results showed that H9C2-shSFRP2 has lower MMP, higher expression levels of FIS1 and DRP1, and lower expression levels of MFN1 and MFN2 than the control group (Figures 5(e)–5(g)). After exposure to the glucolipotoxic milieu, the expression levels of PGC1-*α* and TFAM in the H9C2-shSFRP2 group were significantly lower than those in the control group, while NRF1 was higher than that in the control group (Figure 5(h)). SFRP2 knockdown further aggravated cardiomyocyte apoptosis (Figures 5(i) and 5(j)). Last, SFRP2 knockdown inhibited AMPK phosphorylation (Figure 5(k)). These results further suggested that SFRP2 may be a key molecule to improve mitochondrial function and apoptosis through the AMPK pathway.

### 3.6. Overexpression of SFRP2 Ameliorates Mitochondrial Dysfunction and Apoptosis *In Vivo*

**Figure 6:**
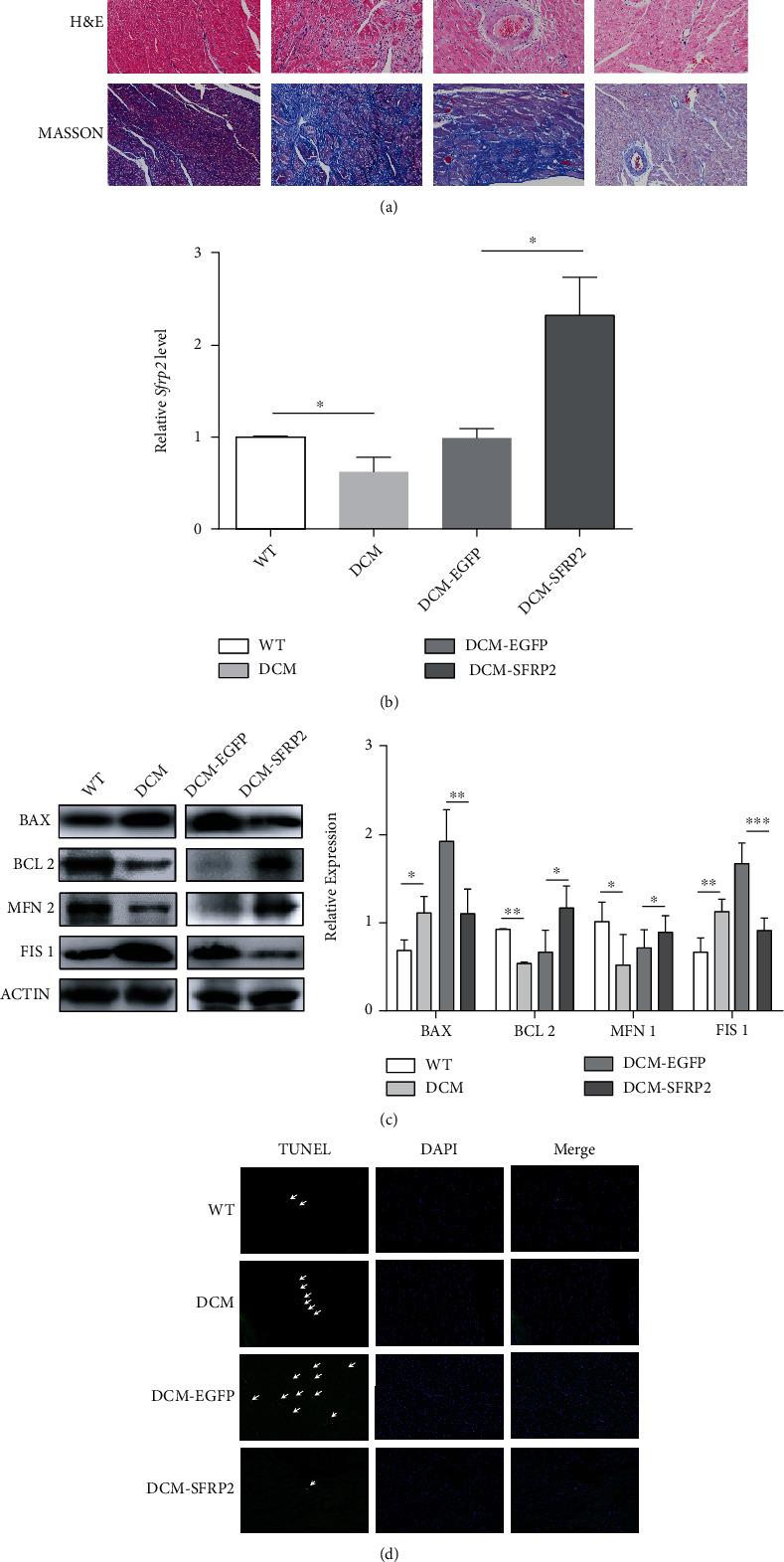
Overexpression of SFRP2 ameliorates mitochondrial dysfunction and apoptosis *in vivo*. (a) Representative images of heart muscle stained with H&E and Masson. (b) The mRNA expression of *Sfrp2* was detected by qRT-PCR. (c) The expression levels of BAX, BCL-2, MFN1, and FIS1 were determined by western blot. (d) Representative images of TUNEL staining show cardiac cell apoptosis. ^∗^*P* < 0.05; ^∗∗^*P* < 0.01; ^∗∗∗^*P* < 0.001.

We next evaluated the therapeutic potential of SFRP2 *in vivo*. After separating the heart tissue, H&E and Masson staining was performed. As shown in Figure 6(a), the cardiac tissues of the wild-type (WT) group were neatly arranged with uniform cell nuclei and only a small amount of collagen fibers was deposited, while the myocardial tissue of the DCM group was disordered, the nucleus size was uneven, and a large amount of collagen fibers was deposited. However, after injection of AAV-SFRP2 through the tail vein, these changes in the myocardium can be reversed. The level of SFRP2 in the DCM group was significantly lower than that in the WT group (Figure 6(b)). Furthermore, DCM rats showed lower BCL-2 and MFN1 levels and higher BAX and FIS1 levels. Consistent with the results of cell experiments *in vivo*, the levels of BCL-2 and MFN1 in the DCM-SFRP2 group were significantly higher than those in the DCM-EGFP group, while BAX and FIS1 were significantly lower than those in the DCM-EGFP group (Figure 6(c)). In addition, we also found that cardiomyocyte apoptosis was exacerbated by diabetes mellitus, while treatment with SFRP2 reduced cardiac cell apoptosis (Figure 6(d)). These data suggested that SFRP2 could reduce cardiac damage and protect cardiac function *in vivo*.

## 4. Discussion

**Figure 7:**
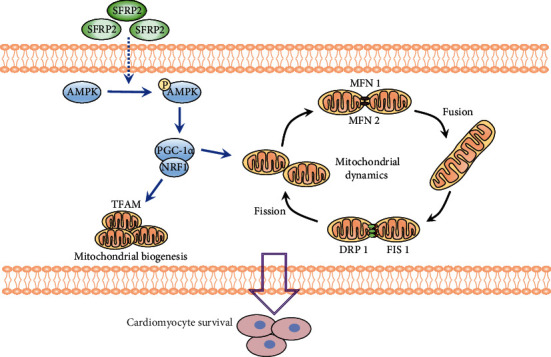
Schematic diagram illustrating that SFRP2 maintains mitochondrial homeostasis and reduces cardiomyocyte apoptosis in hyperglycemia and hyperlipidemia conditions via regulating AMPK-PGC1-*α* signaling. SFRP2 can activate AMPK phosphorylation and furthermore activate PGC1-*α*. PGC1-*α* improves mitochondrial dynamics through regulating mitochondrial dynamics-related proteins and promotes mitochondrial biogenesis activating NRF1 and TFAM.

In this study, we discovered that SFRP2 improves mitochondrial function and quality control through the AMPK-PGC1-α*α* axis, thereby protecting cardiomyocytes against oxidative stress and apoptosis induced by glucolipotoxicity in vitro and in vivo (Figure 7). These beneficial effects of SFRP2 on mitochondria may play a role in hyperglycemia and hyperlipidemia-induced cardiac remodeling and dysfunction.

High glucose can cause oxidative stress and apoptosis of cardiomyocytes [6, 29]. The utilization of glucose by cardiomyocytes decreased in diabetes, which was conducive to the *β*-oxidation of free fatty acids (FFA). In insulin resistance, the lipolysis of adipose tissue is enhanced, leading to an increase in circulating FFA. However, the continuous rise of FFA has a negative effect on myocardial function and eventually leads to increased mitochondrial ROS production and cell apoptosis [30–32]. Corresponding with previous studies, we found that the glucolipotoxic milieu can increase ROS in cardiomyocytes and cause apoptosis *in vitro* and *in vivo* [33–35].

SFRP2 has been reported to have beneficial properties against apoptosis [15, 18, 21, 36] and oxidative stress [21, 36, 37]. Merino et al. found that SFRP2 have valuable therapeutic potential in reversing doxorubicin-induced oxidative stress and apoptosis in soleus muscle [36]. In our previous study, we observed that SFRP2 is an independent biomarker for myocardial fibrosis [18]. In this study, we observed that the expression of apoptosis proteins was decreased, and antiapoptotic protein was increased in type 2 diabetes (T2D) models, accompanied with reduced cell apoptosis in overexpressing SFRP2 cells. The opposite results were observed after knocking down SFRP2. These findings suggested that SFRP2 can improve oxidative stress and apoptosis of cardiomyocytes under the glucolipotoxic milieu. Combined with our early findings, it is more supportive that SFRP2 is a compensatory protective response of heart failure.

The mitochondrial dynamics and mitochondrial biogenesis are very important for maintaining the bioenergy function of mitochondria [38]. The increased fusion or decreased fission promotes the formation of an extended mitochondrial network, and the decreased fusion or increased fission leads to the fragmentation of mitochondria [39]. The imbalance of mitochondrial fusion and fission played a key role in the mechanism for myocardial injury in diabetes mellitus [10, 40]; mitochondrial dysfunction seems to be an important target for therapy to improve cardiac function directly [41]. After abnormal glycolipid exposures, we observed that the mitochondrial membrane potential and intracellular ATP concentration were reduced. Mitochondrial fusion was decreased, while the mitochondrial fission was increased. Simultaneously, the mitochondrial oxidative phosphorylation was impaired in cardiomyocyte [12, 42]. We also found the imbalance of mitochondrial dynamics in T2D rat, but the opposite result was observed in H9C2 cells overexpressing SFRP2 and DCM-SFRP2 rats. These results suggest that SFRP2 can improve mitochondrial function.

PGC1-*α* is a critical regulator of oxidative metabolism, which is involved in maintaining mitochondrial biogenesis and function [43]. Previous studies showed that PGC1-*α* ameliorated cardiac dysfunction and mitochondrial injury in DCM [44, 45], to further prove that PGC1-*α* could activate NRF1 and TFAM to promote mitochondrial biogenesis. Carvedilol, a third-generation and nonselective *β*-adrenoceptor antagonist, could stimulate mitochondrial biogenesis via the PGC1-*α*-NRF1-TFAM pathway and then increase oxygen consumption and mitochondrial respiratory rate in human umbilical vein endothelial cells (HUVEC) [46]. We also observed an increase in PGC1-*α* expression with overexpression of SFRP2, accompanied by an increase in NRF1 and TFAM expression.

AMPK is a key regulator of cardiac energy metabolism, which plays an important role in reducing oxidative stress [47, 48] and antiapoptosis [47, 49, 50] of cardiomyocytes. Additionally, in cultured cardiomyocytes, treatment with recombinant FGF21 counteracted HG-induced oxidative stress, mitochondrial dysfunction, and inflammatory responses, leading to increased AMPK activity expression. However, these beneficial effects of FGF21 were markedly weakened by the genetic blockage of AMPK [51]. Previous findings revealed that AMPK could act as an upstream kinase and activate the expression of PGC1-*α* directly [43]. In our study, we observed that exposure of H9C2 cells to the glucolipotoxic milieu reduced AMPK and PGC1-*α* activity. Overexpression of SFRP2 could activate the AMPK-PGC1a pathway and improve the mitochondrial function of cardiomyocytes under glycolipid toxicity, while SFRP2 was knocked down, the opposite results were observed.

There are several limitations in our study. Firstly, the major findings in our studies were based on cell and rat model. It is still unknown whether they occur in clinical conditions. Secondly, our experiments were particularly focused on the regulatory effects of SFRP2 on mitochondrial dynamics and mitochondrial biogenesis, but we did not pay attention to other functions of mitochondria, such as mitochondrial autophagy. Lastly, we still do not know how SFRP2 regulates the specific mechanism of AMPK, which needs further research.

## 5. Conclusion

Our findings verified the value of SFRP2 in a novel mitochondria-relevant mechanism that mediated cardioprotection via activation of the AMPK-PGC1-*α* signaling pathway in the diabetes-induced cardiac dysfunction. SFRP2 directly regulates the expression of PGC1-*α* through the AMPK signaling pathway and therefore improves the mitochondrial dynamics and mitochondrial biogenesis of diabetic cardiomyocytes and the oxidative stress and apoptosis of cardiomyocytes. These findings identify SFRP2-modulated mitochondrial fusion as a potential target for treating cardiac dysfunction.

## Data Availability

The data used to support the findings of this study are available from the corresponding author upon request.
